# Glutathione Deficiency in *Sinorhizobium meliloti* Does Not Impair Bacteroid Differentiation But Induces Early Senescence in the Interaction With *Medicago truncatula*

**DOI:** 10.3389/fpls.2020.00137

**Published:** 2020-03-03

**Authors:** Li Yang, Sarra El Msehli, Sofiane Benyamina, Annie Lambert, Julie Hopkins, Julie Cazareth, Olivier Pierre, Didier Hérouart, Samira Achi-Smiti, Eric Boncompagni, Pierre Frendo

**Affiliations:** ^1^ Université Côte d'Azur, INRA, CNRS, ISA, Sophia-Antipolis, France; ^2^ Laboratoire de Physiologie Végétale, Faculté des Sciences de Tunis, Campus Universitaire El Manar II, Tunis, Tunisia; ^3^ Institut de Pharmacologie Moléculaire et Cellulaire, CNRS UMR 7275, Université Côte d'Azur, Valbonne, France

**Keywords:** *Medicago truncatula*, *Sinorhizobium meliloti* SmgshB, glutathione, bacteroid differentiation, senescence

## Abstract

Under nitrogen-limiting conditions, legumes are able to interact symbiotically with bacteria of the Rhizobiaceae family. This interaction gives rise to a new organ, named a root nodule. Root nodules are characterized by an increased glutathione (GSH) and homoglutathione (hGSH) content compared to roots. These low molecular thiols are very important in the biological nitrogen fixation. In order to characterize the modification of nodule activity induced by the microsymbiont glutathione deficiency, physiological, biochemical, and gene expression modifications were analyzed in nodules after the inoculation of *Medicago truncatula* with the SmgshB mutant of *Sinorhizobium meliloti* which is deficient in GSH production. The decline in nitrogen fixation efficiency was correlated to the reduction in plant shoot biomass. Flow cytometry analysis showed that SmgshB bacteroids present a higher DNA content than free living bacteria. Live/dead microscopic analysis showed an early bacteroid degradation in SmgshB nodules compared to control nodules which is correlated to a lower bacteroid content at 20 dpi. Finally, the expression of two marker genes involved in nitrogen fixation metabolism, *Leghemoglobin* and *Nodule Cysteine Rich Peptide 001,* decreased significantly in mutant nodules at 20 dpi. In contrast, the expression of two marker genes involved in the nodule senescence, *Cysteine Protease 6* and *Purple Acid Protease*, increased significantly in mutant nodules at 10 dpi strengthening the idea that an early senescence process occurs in SmgshB nodules. In conclusion, our results showed that bacterial GSH deficiency does not impair bacterial differentiation but induces an early nodule senescence.

## Introduction

Nitrogen (N) is the major macronutrient needed for plant growth. However, whereas N is the most abundant element in the atmosphere, it is generally the most limiting plant nutrient in soils. Legume plants are able to interact symbiotically with rhizobia to perform nitrogen fixing symbiosis (NFS). In these mutualistic associations, bacteria deliver nitrogen source to the plants while plants provide bacteria with photosynthates, nutrients required for rhizobium metabolism and a specialized ecological niche that favors their growth. Indeed, the nitrogen-fixing symbiosis involves the *de novo* formation of a new organ, the nodule, associated to a reprogramming of the plant development and metabolism. This allows the intracellular accommodation of several thousands of differentiated bacteria, within nodule cells, called bacteroids which reduce atmospheric nitrogen. Amongst the multiple regulatory processes involved in the setup and the regulation of NFS, a modification of the cellular redox state of the plant partner is observed during the plant infection and nodule functioning (Puppo et al., 2013). Reactive oxygen species production is detected in root hairs in response to nodulation factors (Cárdenas et al., 2008), the root infection process (Santos et al., 2001; Jamet et al., 2007) and in the nodule differentiation zone in *Medicago truncatula* nodules (Andrio et al., 2013). The implication of the ROS in the symbiotic interaction between legumes and rhizobia has been demonstrated using genetic approaches (Marino et al., 2011; Arthikala et al., 2014). Genetic alteration of the expression of respiratory burst oxidative homologs (Rbohs), proteins involved in the ROS production, significantly modifies the symbiosis between legume plants and the rhizobia (Marino et al., 2011; Marino et al., 2012). Similarly, nitric oxide (NO) production is also detected in the NFS (Hichri et al., 2015). It is required for the optimal establishment of the symbiotic interaction (Hichri et al., 2015; Berger et al., 2019). In mature nodules, NO has a beneficial metabolic function for the maintenance of the energy status under hypoxic conditions, but has also been shown to inhibit nitrogen fixation and to trigger nodule senescence. NO has also been shown to regulate the glutathione (GSH) synthesis in roots (Innocenti et al., 2007).

The root nodules contain significantly higher contents of low molecular thiols, GSH and homoglutathione (hGSH), in comparison to roots (Frendo et al., 2013a; Frendo et al., 2013b). GSH is the most abundant low-molecular weight thiol in cells and has several well-known functions. For example, it acts as a redox buffer and as a signaling molecule, and it is also involved in the detoxification of radicals and xenobiotics (Rouhier et al., 2008; Foyer and Noctor, 2011). GSH is synthesized by a two-step process. In the first step, glutamate and cysteine are conjugated by γ-glutamyl cysteine synthetase (γ-ECS) to form γ-glutamyl cysteine (γ-EC). In a second step, glycine is added to γ-EC to form GSH in a reaction catalyzed by glutathione synthetase (GSHS). Alternatively, homoglutathione synthetase (hGSHS) catalyzes the addition of β-alanine to γ-EC to form hGSH. Genetic and pharmacological approaches showed that the GSH pool produced by the plant partner is important to allow the nodule development and an efficient biological nitrogen fixation (BNF) (Frendo et al., 2005; El Msehli et al., 2011). On the bacterial side, the use of mutants deficient in the GSHS-encoding gene (*gshB*) impairs the BNF efficiency during the symbiosis between *Medicago sativa*/*S. meliloti* strain 1021, *Phaseolus vulgaris/Rhizobium tropici*, *P. vulgaris/Rhizobium etli,* and *Pisum sativum*/*Rhizobium leguminosarum* (Harrison et al., 2005; Muglia et al., 2008; Taté et al., 2012; Cheng et al., 2017). The reduced BNF observed with the *gshB* mutant strain was correlated with nodule and bacteroid early senescence in *M. sativa and P. vulgaris* (Harrison et al., 2005; Muglia et al., 2008; Taté et al., 2012). In parallel, recycling of GSH by glutathione reductase was shown to be required to maintain an effective nitrogen fixation (Tang et al., 2018). Finally, a deficiency in bacteroid differentiation was observed for a *S. meliloti* (strain 2011) mutant for glutaredoxin 1 (Grx1), a glutaredoxin involved in the deglutathionylation of proteins using GSH as a cofactor, in the symbiotic interaction with *M. truncatula* (Benyamina et al., 2013). Harrison and colleagues (2005) also suggested that glutathione deficiency in *S. meliloti* strain 1021 interferes with bacteroid differentiation *in M. sativa* (Harrison et al., 2005) but this hypothesis was not carefully investigated.

In this context, we hypothesized that the genome background of *S. meliloti* (strain 1021 versus strain 2011) or the genome background of the plant (*M. sativa* versus *M. truncatula*) could modify the outcome of the interaction between the two partners. With this in mind, we intended to characterize the impact of the bacterial GSH deficiency on the bacteroid differentiation and the nodule functioning during the symbiotic interaction between *M. truncatula* and the strain 2011 of *S. meliloti*. Physiological, biochemical, cellular and genetic markers were used to describe the nodule functioning at 10 and 20 days after plant inoculation, two early time points of nodule functioning. Our results show that bacterial GSH deficiency does not affect bacteroid differentiation. However, bacterial GSH deficiency induces an early nodule senescence process in *M. truncatula* already detectable at 10-d post-infection.

## Materials and Methods

### Plant Material, Bacterial Strains and Growth Conditions

A mutation in *gshB* (Smc00419) of *S. meliloti* 2011 was made by plasmid integration using single-crossover. A 680-bp *gshB* fragment was PCR-amplified using primers SMc00419-F (5'-AAGAATTCCCATGTTTCGGGCATCACC-3') and SMc00419-R (5'-AACGATC GCTGCAGTGCATGTTGGAGCGAGAATC-3'). The fragment was cloned into *Eco*RI-*Pvu*I sites of pSUP202, resulting in plasmid pSUP202gshB. Plasmid pSup202gshB was transferred from *E. coli* to *S. meliloti* 2011 and recombined into the genomic *gshB* region *via* single crossover to give the mutant strain SmgshB in *S. meliloti* 2011 genetic background. The mutants were screen for resistance to tetracyclin (10 μg per ml) and streptomycin (100 μg per mL). The SmgshB mutant strain in Sm 2011 genetic background was validated by HPLC for the lack of GSH as described previously (Harrison et al., 2005). The delayed growth phenotype observed for the mutant strain was complemented by the addition of GSH (**Figure S1**). *M. truncatula* ecotype *Jemalong* A17, *S. meliloti* 2011 (wild type), and *S. meliloti* 2011 gshB (SmgshB) strains were grown as described (Harrison et al., 2005). *M. truncatula* seeds were then spread on 0.4% agar gel at 4°C for 2 days for vernalization and germinated at 16°C for 1 day. Seedlings were afterwards growing *in vitro* on Fahraeus medium (Boisson-Dernier et al., 2001). Each seedling was inoculated with 200µl of *S. meliloti* at an O.D._600nm_ of 0.05. All the plants used in this study were grown in a 16-h light (23°C): 8-h dark (20°C) photoperiod. Nodules were harvested at 10- and 20-days post-inoculation (dpi). At least three independent biological repetitions were analyzed.

#### Nitrogenase Activity Assay

Nitrogen fixation activity was determined by C_2_H_2_ reduction assay (ARA), using a gas chromatograph. Nodulated roots at 10 and 20 dpi were detached from plants and put inside sealed vials. In each vial, 10% of the atmosphere was substituted with acetylene. After 1 h of incubation at 28°C, gas samples were analyzed with a gas chromatograph system (Agilent GC 6890N; Agilent Technologies, Massy, France). Twelve plants were tested per replicate and at least three different biological replicates were analyzed.

#### Flow Cytometry

*S. meliloti* bacteroids were purified as previously described (Trinchant et al., 2004) using a 320 mOsM extraction buffer. Bacterial cells were fixed by heat treatment (70°C, 15 min) and stained with 10 μg/mL propidium iodide (PI). They were then subjected to flow cytometry analysis with a LSRII Fortessa flow cytometer (BD BioSciences, Rungis, France). Bacterial DNA content was determined from PI fluorescence at 585-625 nm after excitation at 565 nm with a single laser line. Single events were recorded and analyzed with BD FACSDiva v6.1.3 (BD BioSciences) and Kaluza v1.2 software (Beckman Coulter, Villepinte, France), respectively. Experiments were performed with biological triplicates and the recording of more than 200,000 events for each sample to ensure the statistical robustness of each assay (Ribeiro et al., 2017).

#### Live/Dead Staining and Microscopic Observations

Nodulated roots and nodules at 10 and 20 dpi, respectively, were harvested and embedded in 4% agarose. Nodule sections of 70µm were prepared with a Vibratome HM650V (Thermo Fisher Scientific, Illkirch-Graffenstaden, France). Afterwards, nodule sections were stained with SYTO^™^ 9 and propidium iodide (PI) (LIVE/DEAD BacLight Bacterial Viability Kit, Thermo Fisher scientific) for 20 min as previously described (Haag et al., 2011). Sections were then mounted on slides and visualized with a laser scanning confocal microscope LSM880 (Carl Zeiss, Marly le Roi, France). The SYTO™ 9 and PI were sequentially excited by 488 nm Argon and 561 nm HeNe laser lines, separately. Both fluorescence signals were recorded on separated detectors. All images were treated with Fiji software (Schindelin et al., 2012). Samples are from at least 30 nodules of 15 plants from three independent replicates.

#### Quantitative Reverse Transcriptase PCR (RT-qPCR)

Young nodules (10 dpi) and mature nodules (20 dpi) were harvested. Total RNA was extracted from 100 mg of nodules with Trizol (Invitrogen, Illkirch-Graffenstaden, France) and cDNA was synthesized with the Omniscript RT kit (Qiagen, Les Ulis, France) after a DNAse treatment of total RNA. Absence of genomic contamination was ensured by control PCR with intron-spanning primers. Quantitative PCR was performed using AriaMx Real-time PCR system (Agilent, Les Ulis, France). All data were normalized with two constitutively expressed genes, *Mtc27* and *A38* (*M. truncatula*) for the *M. truncatula* genes, and *16S ribosomal RNA* and *Polyribonucleotide nucleotidyltransferase* for the *S. meliloti* genes. The primer sequences used for PCR are described in **Table S1**. Each RT-qPCR reaction for each of the three biological experiments was performed in triplicate. The expression fold change was calculated as 2^-∆∆Ct^ method (Livak and Schmittgen, 2001).

#### Statistical Analyses

Our data were reported as mean ± standard error. The significance of the results was assessed using the Mann–Whitney nonparametric test, which allows the comparison of small quantitative samples. When the number of samples was enough to define a normal distribution (30 samples), the results were assessed using Student's t-test.

## Results

### Analysis of Plant Phenotype and Biological Nitrogen Fixation

**Figure 1:**
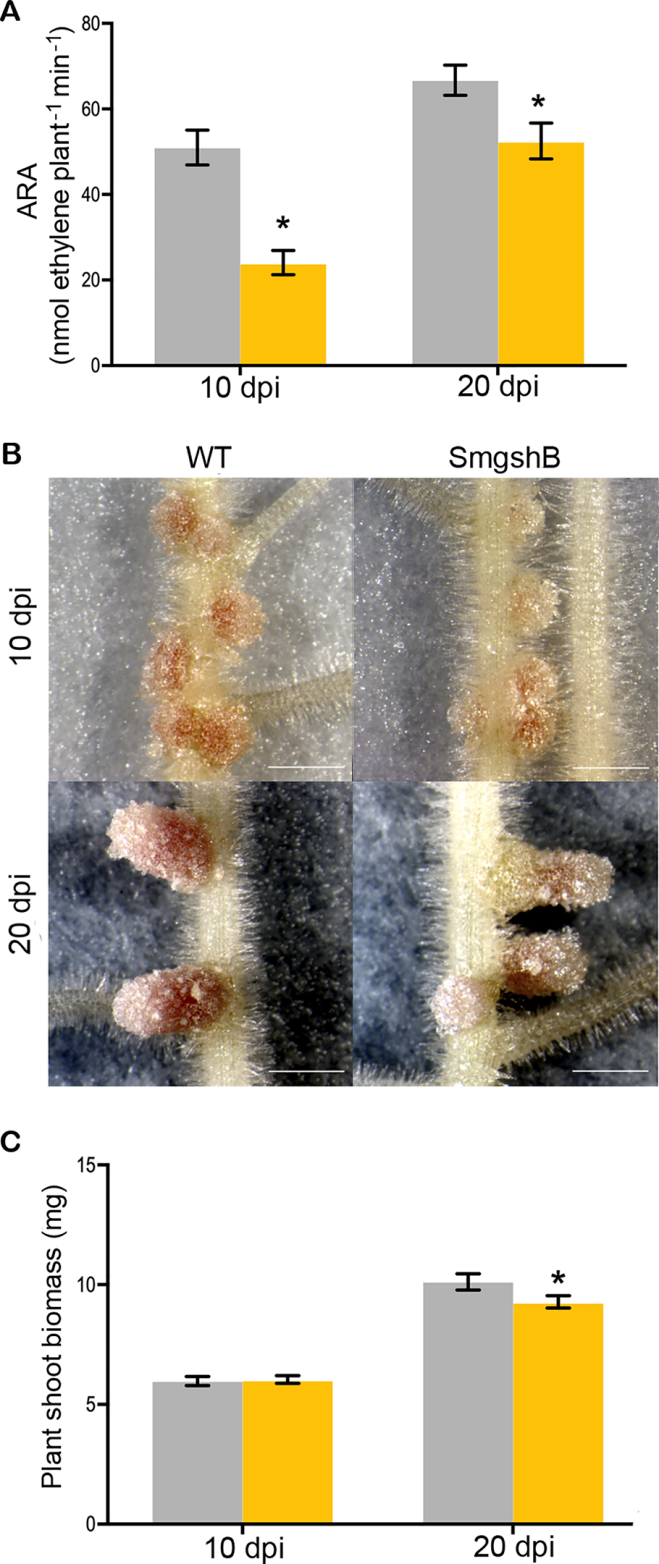
Physiological analysis of the symbiosis between *Medicago truncatula and Sinorhizobium meliloti* SmgshB mutant strain. **(A)** Nitrogen fixation capacity of plants with WT strain (grey) and the mutant SmgshB (yellow) at 10- or 20-d post-inoculation (dpi), measured by the acetylene reduction assay per plant (nanomoles of ethylene produced per minute of incubation and per plant). **(B)** Phenotype of nodules formed with indicated strains at 10- or 20- dpi. Scale bars = 1 mm. **(C)** Dry weight shoot mass of *M. truncatula* inoculated with WT (grey) and SmgshB mutant (yellow) strains at 10 or 20 dpi. Error bars are standard errors and * indicates a statistically significant difference (P < 0.05; n = 15).

To test the phenotype of the *S. meliloti* SmgshB strain, we have analyzed nodule functioning at 10- and 20- dpi which corresponds to young and mature nodules *in vitro* growth conditions. The phenotypes of the plants and the root nodules were examined, and the nitrogen fixation activity was analyzed by ARA (**Figure 1**). The nitrogen fixation efficiency was significantly reduced by 53% and 21% in SmgshB inoculated plants compared to control plants at 10 and 20 dpi, respectively (**Figure 1A**). A significant lower number of nodules was observed for SmgshB inoculated plants compared to the control plants at 10 dpi (**Figure S2**) and nodule size was not different at 10 or 20 dpi (**Figure S2**). Phenotypic analysis of root nodules showed that SmgshB nodules presented a less pronounced pink color compared to control ones and present an extended green color on the distal part of nodule at 20 dpi (**Figure 1B**). A significant diminution of shoot dry weight biomass and a reduced plant development were observed in SmgshB inoculated plants compared to the control plants at 20 dpi (**Figures 1C** and **S3**). In conclusion, SmgshB inoculated plants present an altered growth phenotype compared to the control plants.

### Analysis of Bacteroid Differentiation Using Flow Cytometry

**Figure 2:**
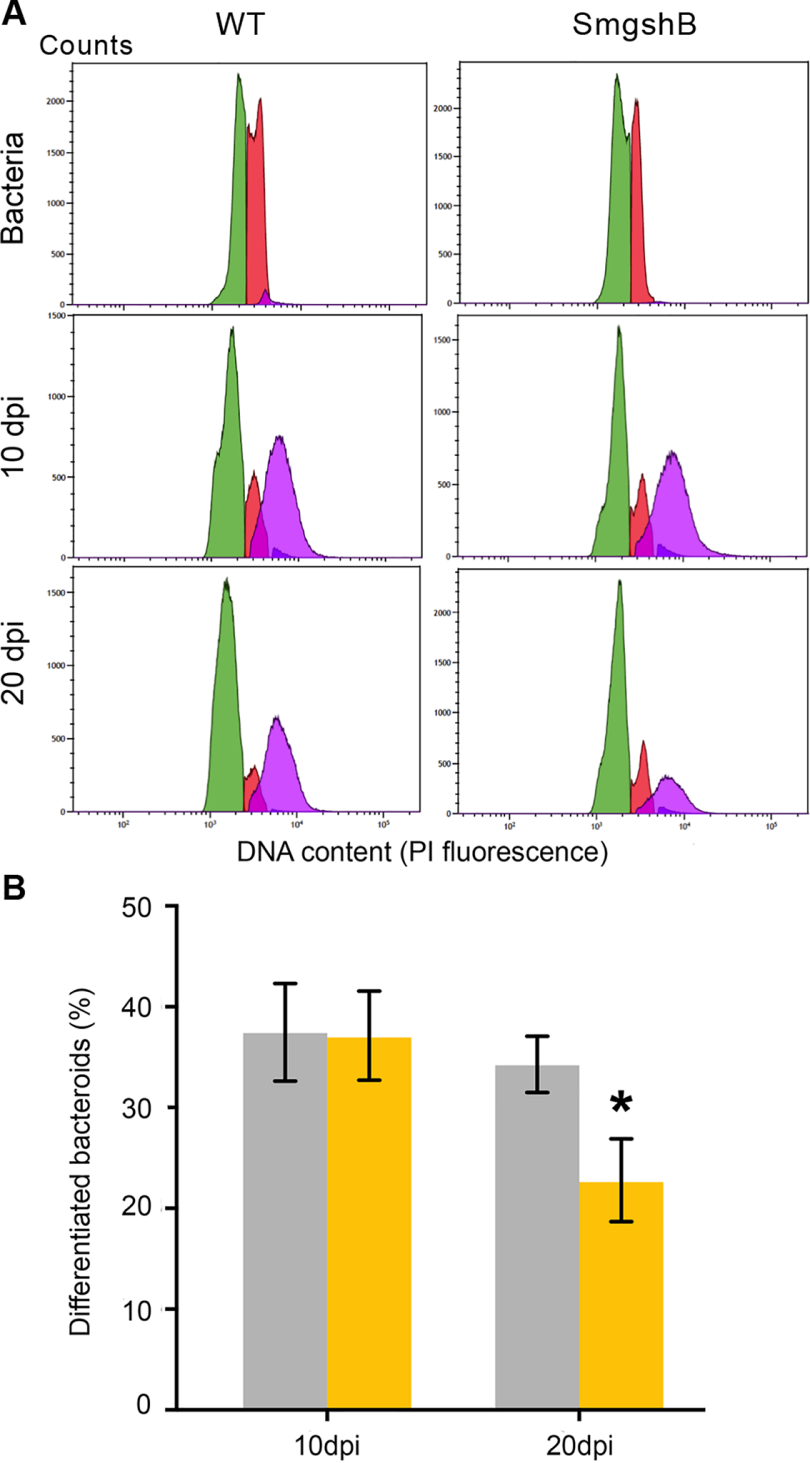
Flow cytometry analysis of SmgshB bacteroids. **(A)** DNA content (PI florescence) of bacteria and bacteroids extracted from nodules obtained with *S. meliloti* 2011 strain or SmgshB mutant strain after 10 or 20 dpi. DNA content of bacterial and bacteroid populations is indicated in color as: green: 1C, red: 2C, purple: 4C and more. The datasets shown are representative of trends observed in three independent experiments. **(B)** Percentage of bacteroids with a large SSC (side scatter) value and DNA value of 4C and more. The values shown are the means ± SE of three independent replicates (at least two plants per replicate). Signiﬁcance was determined using the Mann–Whitney nonparametric test (P < 0.05; n = 3). The * indicates a statistically significant difference (P < 0.05; n = 3).

The bacteroid differentiation is correlated to an increase in size and in DNA content of the bacteroid compared to the bacterium (Mergaert et al., 2006). These criteria could be used to differentiate bacteria from bacteroids using flow cytometry (Ribeiro et al., 2017). To test whether GSH deficiency in the bacteria impairs the bacteria differentiation, we have analyzed the bacteroid and bacterial content in nodules from control plants and SmgshB inoculated plants at 10 and 20 dpi using flow cytometry (**Figure 2**). Bacterial cells showing DNA endoreduplication were observed in both control and SmgshB nodules at 10 and 20 dpi (**Figure 2A**). The nitrogen-fixing bacteroid (DNA content 4C and more) proportion in control and SmgshB nodules was similar at around 40% at 10 dpi (**Figure 2B**). In contrast, the proportion of bacteroids was significantly lower in SmgshB nodules than in control ones at 20 dpi, with a reduction of 40% in bacteroid content compared to the control (**Figure 2B**). Therefore, these results show that bacteroid differentiation occurs in SmgshB nodules in a similar way that in control nodule at 10 dpi. However, the lower content of bacteroids in SmgshB nodules compared to control ones shows that the nodule bacteroid content decreases in mature nodules. This decrease of bacteroid content in SmgshB nodules may be associated to a reduced bacterial differentiation during nodule aging or to a faster bacteroid degradation compared to control nodules.

### Analysis of Bacteroid Survival Using Live/Dead Staining in Nodule

**Figure 3:**
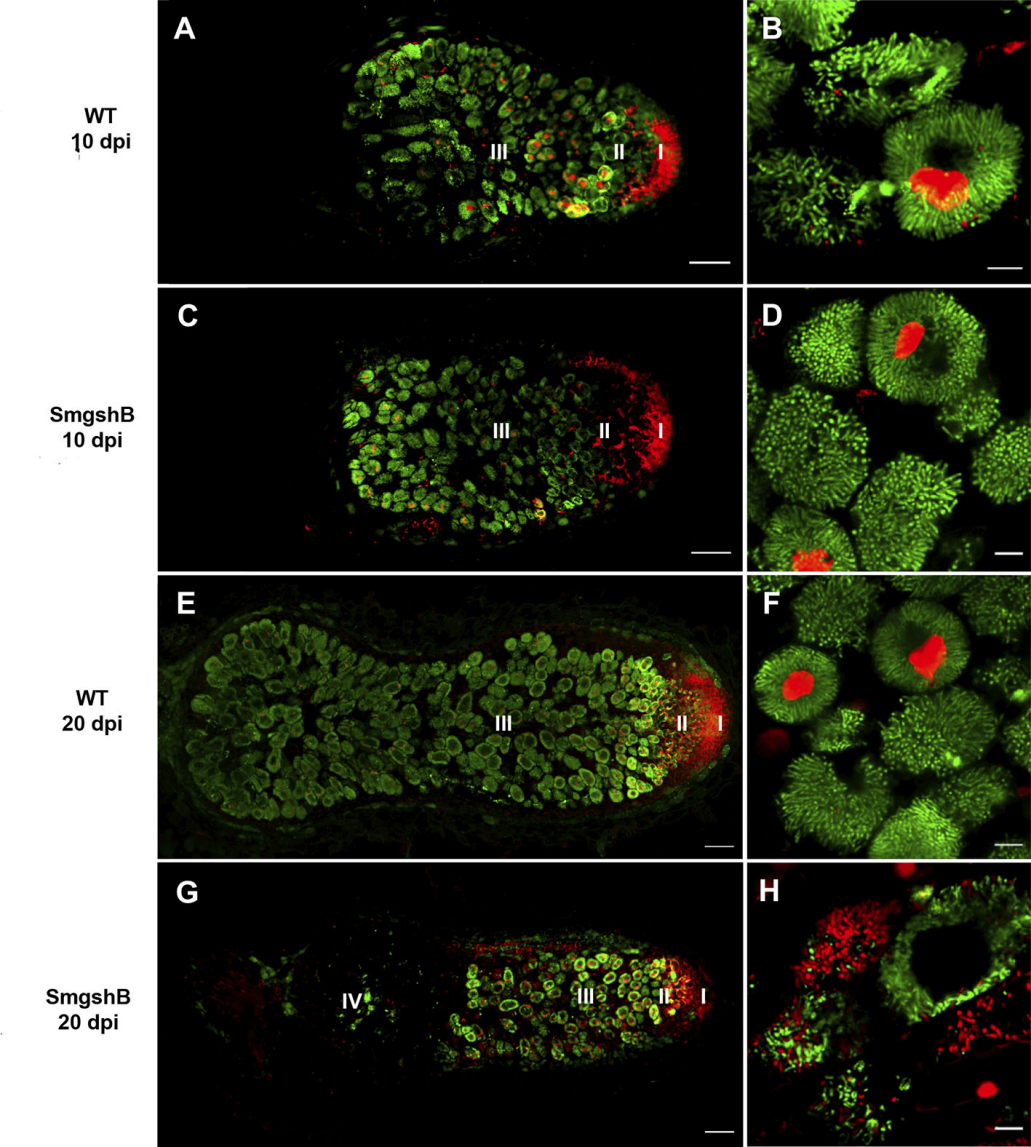
Live/dead microscopic analysis of SmgshB nodules. *M. truncatula* plantlets were inoculated with either WT or SmgshB strains. Bacteroid viability of nodule sections determined live/dead staining by SYTO9 and PI dyes at 10 and 20 dpi. Viable bacteria/bacteroid are stained green by SYTO9 and damaged bacteroids are stained red by PI. The different zones of the nodule are indicated: I, the meristem; II, the infection zone; III, the nitrogen fixation zone; and IV, the senescent zone. Panel **(B, D, F, H)** represent infected cells in zone III. The images shown are representatives of at least 30 nodules of 15 plants from three independent replicates. Scale bars = 100 µm **(A,** **C,** **E,** **G)** or = 10 µm **(B,** **D,** **F,** **H)**.

To analyze the survival of bacteroids in the SmgshB nodules, the live/dead staining was used at 10 and 20 dpi (**Figure 3**). Permeable bacteroid cell membranes that are considered dead or dying and plant nucleus are stained red with propidium iodide (PI), while bacteroids with intact membranes are green due to SYTO^™^ 9 staining of nucleic acids (Haag et al., 2011). No significant difference was observed at 10 dpi between the SmgshB and control bacteroids (**Figures 3A, C**). At 20 dpi, the nodules are more elongated with a larger zone III in control nodules. No significant bacteroid degradation was observed in the control nodules (**Figure 3F**). In contrast, SmgshB nodules presented a strong alteration of the nodule cellular structure with numerous dead bacteroids and empty plant cells in zone III (**Figure 3H**). These results show that the cellular structure of SmgshB nodule is not modified at early time point but present significant degradation at 20 dpi. This suggests that senescence occurs ealier in SmgshB nodules than in control nodules.

### Expression of Nodule Marker Genes in SmgshB Nodules

**Figure 4:**
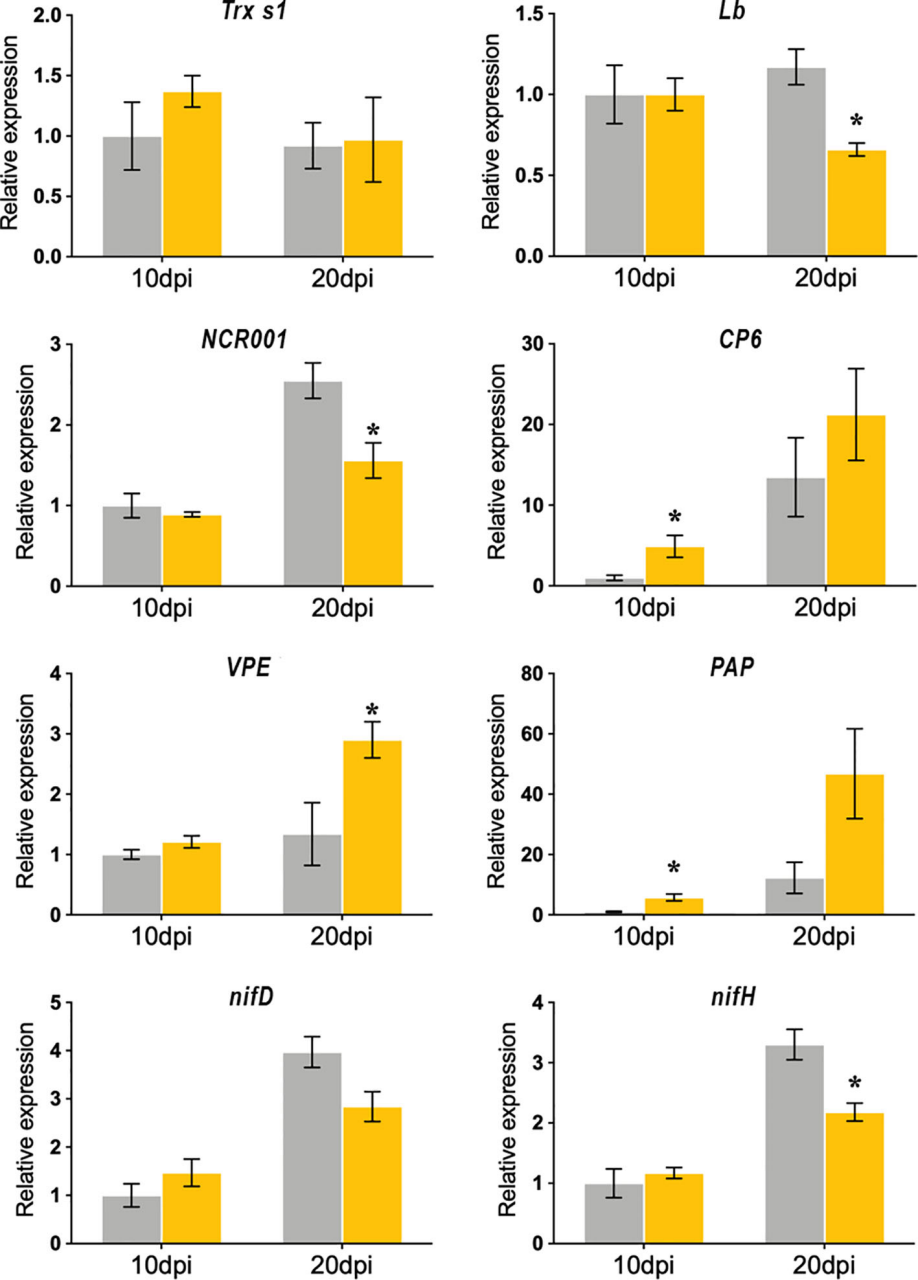
Quantitative RT-PCR analysis of nodule gene expression markers in *M. truncatula* nodules inoculated with WT (grey) and SmgshB strains (yellow). The expression levels of *Nodule Cysteine Rich 001* (*NCR001*), *Leghemoglobin* (*Lb*), *Thioredoxin s1* (*Trx s1*), *Cysteine Protease 6* (*CP6*), *Vacuolar Processing Enzyme* (*VPE*), *Purple Acid Phosphatase* (*PAP*), *nifD* (nitrogenase molybdenum-iron protein alpha chain) and *nifH* (nitrogenase iron protein). were analyzed in nodules. Data (technical triplicates of three biological samples) are reported as mean ± standard error. * indicates a statistically significant difference relative to the control (P < 0.05; n = 3).

In order to analyze whether the cellular and biochemical alterations observed in the SmgshB nodules could be correlated to modifications in the nodule genetic program, the expression level of plant gene markers was analyzed by RT-qPCR. These genes are involved either in the functioning of nodules or are genes potentially markers of senescence. The expression of eight marker genes was measured to analyze the SmgshB nodule transcriptomic modifications (**Figure 4**). The expression of marker genes expressed in the nodule differentiation zone (*Thioredoxin s1*, *Trx s1*), in the nitrogen fixation zone (*Nodule Cysteine Rich Peptide, NCR001* and *leghemoglobin, Lb*) and during nodule senescence (*Cysteine Proteases 6, CP6*, *Vacuolar Processing Enzyme*, *VPE*, and *Purple Acid Protease*, *PAP*) was studied in control and SmgshB nodules. Moreover, the expression of two bacterial genes *nifD* (nitrogenase molybdenum-iron protein alpha chain) and *nifH* (nitrogenase iron protein), encoding nitrogenase subunits and expressed in nitrogen-fixing bacteroids, was analyzed in control and SmgshB nodules. *Trx s1* is specifically expressed during the symbiotic interaction between *M. truncatula* and *S. meliloti* (Alkhalfioui et al., 2008) and its expression is localized in the nodule infection zone (Ribeiro et al., 2017). *NCR001* is considered as a specific marker of the nitrogen fixation zone (Mergaert et al., 2003) like *Leghemoglobin* (Mergaert et al., 2003; Ott et al., 2005). The expression of *CP6* and *VPE,* two genes encoding cysteine proteases, is induced during developmental- and stress-induced nodule senescence (Pierre et al., 2014). Finally, the expression of *PAP* is induced during the nodule senescence process in *M. truncatula* (Xi et al., 2013). *In silico* analysis of this *PAP* isoform showed that it is significantly more expressed in the nodule than in roots and significantly more expressed in the nitrogen fixation zone than in the nodule meristematic and differentiation zones (**Table S2**).

Analysis of *Trx s1* expression showed that *Trx s1* expression was not significantly modified in SmgshB nodules compared to control nodules at 10 and 20 dpi suggesting a normal functioning of the nodule infection zone. The expression levels of *NCR001* and *Lb* are significantly down-regulated in SmgshB nodules in comparison to control ones at 20 dpi. The expression of *nifH* and *nifD* was similar in SmgshB and control nodules at 10 dpi and *nifH* was significantly down-regulated in SmgshB nodules in comparison to control ones at 20 dpi. Regarding the senescence related gene markers, *CP6, VPE,* and *PAP* expression are, conversely, significantly up-regulated in SmgshB nodules compared to control nodules. The similar expression of *nifH* and *nifD* at 10 dpi strengthens the flow cytometry analysis and shows that the SmgshB bacteria are able to differentiate into bacteroids. Moreover, the down-regulation of the genes involved in BNF efficiency is correlated to the induction of senescence related genes, confirming that an early senescence occurs in SmgshB nodules.

## Discussion

The symbiotic interaction established between legumes and bacteria allows the plant to satisfy its needs in nitrogen. The use of a bacterial strain deficient in GSH production alters significantly the biological nitrogen fixation and the growth of *M. sativa, P. vulgaris,* and *P. sativum during NFS (* Harrison et al., 2005; Muglia et al., 2008; Taté et al., 2012; Cheng et al., 2017 *)*. Moreover, the electronic microscopy analyses suggested that SmgshB mutant strain is partially impaired in the bacteroid differentiation (Harrison et al., 2005). More recently, the glutaredoxin SmGrx1 mutant in *S. meliloti* strain 2011 showed clearly a deficiency in bacteroid differentiation. Glutaredoxins (Grxs) are small ubiquitous redox enzymes that are involved in the reduction of oxidative modifications using GSH (Benyamina et al., 2013). Thus, our hypothesis is that GSH produced by the strain 2011 is necessary to allow normal bacteroid differentiation in nodules formed on *M. truncatula*. In this context, we tested the symbiotic interaction between *M. truncatula* and the GSH deficient-SmgshB mutant constructed in *S. meliloti* strain 2011 at 10 and 20 dpi, two early time points of nodule nitrogen fixation, using a multidisciplinary approach.

The nitrogen fixation efficiency was significantly impaired in plants inoculated with SmgshB strain (**Figure 1**). However, this reduction is less noticeable than in plants inoculated with SmGrx1 (Benyamina et al., 2013). Moreover, at 20 dpi, elongated nodules are observed suggesting that the nodule meristem is more active in plants inoculated with SmgshB strain than in plants inoculated with SmGrx1 strain (Benyamina et al., 2013). In *M. truncatula*, the bacteroids present a terminal differentiation accompanied with a DNA endoreduplication and an arrest in cell division coupled to an increase in cell size (Ribeiro et al., 2015). The flow cytometry analysis of SmgshB bacteroids shows that DNA endoreduplication occurs in SmgshB bacteroid and no significant difference was observed between SmgshB nodules and control ones at 10 dpi (**Figure 2**). In contrast, the number of terminally differentiated bacteroids is significantly lower in SmgshB nodules than in control ones at 20 dpi suggesting that there is a faster degradation or a slower formation of bacteroids in SmgshB nodules than in control ones. The live/dead experiments allow us to analyze the viability of the bacteroids in the root nodules. Such live/dead labelling shows an early death of bacteroids in SmgshB nodules compared to control ones at 20 dpi (**Figure 3**).

Gene expression analysis showed that at 10 dpi, genes expressed in the nitrogen fixing zone, *NCR001* and *Lb*, are similarly expressed in SmgshB and control nodules suggesting that the gene expression in the nitrogen fixing zone is not significantly affected (**Figure 4**). A similar result is observed for *Trx s1*, a gene marker for the nodule infection zone, and *VPE*. Similarly, expression of *nifH* and *nifD* genes was similar in SmgshB and control nodules strengthening that SmgshB bacteroid differentiation occurs properly at 10 dpi. In contrast, significant higher expression of *CP6* and *PAP* is already observed in SmgshB nodules compared to control ones at 10 dpi. This result showed that in our plant growth conditions, *CP6* and *PAP* are the earlier senescence marker genes whose expression is significantly modified, suggesting that there is an early development of senescence in SmgshB nodules. At 20 dpi, there is a significant modification in the expression of different genes. *NCR001*, *Lb*, and *nifH* are less expressed in SmgshB nodules compared to control ones showing a correlation between the diminution of the nitrogen fixation efficiency and the reduction of the expression of genes expressed in the nitrogen fixation zone. Despite a strong increase of *CP6* expression in 20 dpi mature SmgshB nodule, there is no more a significant difference compared to the control suggesting that a senescence process begins in control nodules in our conditions. In contrast, *VPE* expression is significantly induced in SmgshB nodule indicating the progression of the senescence at 20 dpi as observed in the live/dead analysis.

Taken together, our data show that, in contrast to SmGrx1 bacterial mutant (strain 2011), SmgshB mutant (strain 2011) is able to differentiate in bacteroids inside *M. truncatula* cells. The phenotype observed is similar to the one obtained during the interaction of SmgshB mutant (strain 1021) with *M. sativa (* Harrison et al., 2005 *).* In this context, SmGrx1 should use another reducing power than glutathione to perform its activity. We hypothesize that the Grx1 could use the γ-EC accumulated in high quantity in the SmgshB mutant strain as cofactor for the reductive power instead of GSH. Different phenotypes were observed for *Arabidopsis thaliana* γ-ECS (*gsh1*) and GSHS (*gsh*2) plant mutants. The ghs1 mutant presents an embryo lethal phenotype whereas the gsh2 mutant has a seedling lethal phenotype suggesting a partial replacement of GSH functions by γ-EC (Cairns et al., 2006; Pasternak et al., 2008). A similar observation is also true for *S. meliloti* strain 1021 in which the SmgshA mutant strain cannot divide while the SmgshB mutant strain shows a growth delay under free living conditions *(* Harrison et al., 2005 *)*. γ-EC is also used as a cofactor by glutathione peroxidase-1 to detoxify ROS in mice (Quintana-Cabrera et al., 2012).

**Figure 5:**
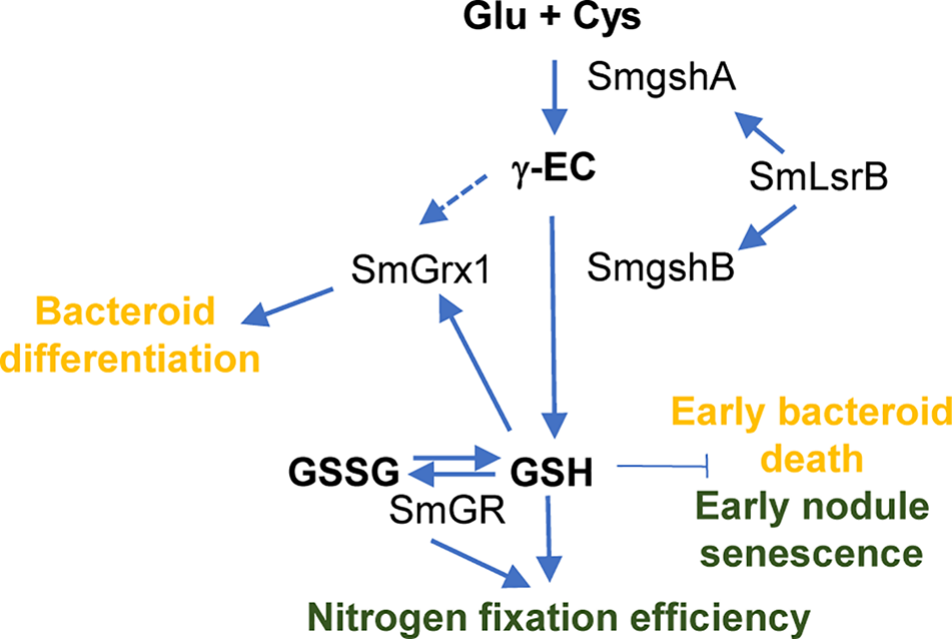
General overview of GSH role in *S. meliloti* bacteroid. GSH plays a central role in the redox networks of glutaredoxin (SmGrx1) and SmLsrB. Defect in GSH accumulation lead to an early nodule senescence whereas deletion in Grx1 impairs bacteroid differentiation. The green color indicates general effects on the root nodule and the orange color indicates direct effects on the bacteroid. Dashed arrow indicates the potential role of γ-EC on SmGrx1 in absence of GSH.

The correlation between the GSH content and the BNF in various legumes was the object of several works (Dalton et al., 1986; Evans et al., 1999; Matamoros et al., 1999; Moran et al., 2000; Groten et al., 2005). The plant GSH content is crucial for the bacterial infection effectiveness and the nitrogen fixation efficiency. GSH produced in the plant does not compensate the *gshB* mutation suggesting that it is not able to be transported to the bacteroid (Becana et al., 2018). In this context, it should be mentioned that sulfate transport from the plant cell to the bacteroids is very active (Schneider et al., 2019) and that mutation in the sulfate transporter *SST1* in *Lotus japonicus* impairs symbiotic nitrogen fixation (Krusell et al., 2005). These data suggest that the sulfur metabolism in bacteroid is depending on sulfate transport and that other sulfur containing molecules such as methionine, cysteine or GSH are not transported through the symbiosome membrane. In this sense, the *R. etli metZ* gene involved in methionine synthesis is required for the efficient interaction with *P. vulgaris* suggesting that methionine is produced by *R. etli* bacteroids during symbiosis, and not supplied by the host cells (Taté et al., 1999). In contrast, the *cysG* mutant strain of *R. etli*, unable to grow on sulfate as the sole sulfur source, was able to induce nitrogen-fixing nodules on *P. vulgaris* suggesting that cysteine or glutathione is supplied by the plant to bacteria (Taté et al., 1997). Moreover, the nodules induced by cysteine auxotrophs of *S. meliloti* Rmd201 were fully effective like those of the parental strain-induced nodules suggesting that the alfalfa host provides cysteine to the bacteroid (Abbas et al., 2002). However, even if gshB mutant strains show an impaired symbiotic phenotype, it cannot be excluded that a small fraction of GSH produced in the plant may pass through the bacteroid membrane, partially compensating the bacterial deficiency. The bacterial GSH pool plays also a crucial role for nitrogen fixation efficiency (**Figure 5**). In the gshB mutant of *S. meliloti* strain 1021, the GSH deficiency was associated with a higher catalase activity in the bacterial mutant in free-living conditions and a reduction of growth efficiency *(* Harrison et al., 2005 *).* A decrease in the BNF was observed during the symbiosis with *M. sativa* correlated to an early bacteroid death. In *R. tropici gshB* mutant strain, the lower symbiotic efficiency was associated to an early senescence pattern connected to increased levels of superoxide accumulation (Muglia et al., 2008). For *Rhizobium etli gshB* mutant, the decrease in BNF was correlated to a strong reduction in nodule number and a deficiency of glutamine transport through Aap and Bra transporter (Taté et al., 2012). This deficiency in nutrient transport also detected in *R. leguminosarum* 3841, which fails to efficiently use various carbon sources such as glucose, succinate, glutamine and histidine, may trigger an early nodule senescence. This transport deficiency in *R. leguminosarum* 3841 *gshB* mutant strain is also associated to a lower BNF (Cheng et al., 2017). The LysR-type transcriptional regulator LsrB, required for nodulation, also regulates *gshA* gene expression through modification of cysteine residues (Luo et al., 2005; Tang et al., 2018) and *gshB* gene expression (Lu et al., 2013).

In conclusion, the inoculation of *M. truncatula* with the SmgshB bacterial mutant triggers the development of nodules with a reduced capacity to fix nitrogen. This lower BNF does not seem correlated to a reduced bacteroid differentiation efficiency. However, it is strongly correlated to an early senescence process and the alteration of the expression of multiple nodule gene markers showing the fundamental role of the bacterial GSH pool in nodule functioning.

## Data Availability Statement

The datasets generated for this study are available on request to the corresponding author.

## Author Contributions

Conceived and designed the experiments: LY, SE, EB, PF. Performed the experiments: LY, SE, SB, AL, JH, JC, OP, EB. Analyzed the data: LY, SE, EB, PF. Wrote the paper: LY, SE, EB, PF. Amended the paper: SAS, DH.

## Funding

LY is supported by a doctoral fellowship from the China scholarship council (CSC). SE was financed by IMAGEEN program from the European Communities and the Tunisian government (bourse d'alternance). This work was supported by the “Institut National de la Recherche Agronomique”, the “Centre National de la Recherche Scientifique”, the University of Nice-Sophia Antipolis and the French Government (National Research Agency, ANR) through the “STAYPINK” project (reference # ANR-15-CE20-0005) and the “Investments for the Future” LABEX SIGNALIFE: program reference # ANR-11-LABX-0028-01.

## Conflict of Interest

The authors declare that the research was conducted in the absence of any commercial or financial relationships that could be construed as a potential conflict of interest.
