# Supplementary material for: Glutathione Deficiency in Sinorhizobium meliloti Does Not Impair Bacteroid Differentiation But Induces Early Senescence in the Interaction With Medicago truncatula
Source: Front Plant Sci. 2020 Mar 3;11:137. doi: 10.3389/fpls.2020.00137 (PMC7063052; doi:10.3389/fpls.2020.00137)
Supplement: Supplementary file 1 [file DataSheet_1.docx]

**Table S1**: List of primers used to quantify gene expression levels by qRT-PCR

| **Genes** | **Forward primers** | **Reverse primers** |
| --- | --- | --- |
| ***Mtc27*** | TGAGGGAGCAACCAAATACC | GCCAAAACCAAGCTACCATC |
| ***A38*** | TCGTGGTGGTGGTTATCAAA | TTCAGACCTTCCCATTGACA |
| ***16S*** | GATAAGCCGAGAGGAAGGTG | GTGTAGCCCAGCCCGTAAG |
| *16S ribosomal RNA* |  |  |
| ***PNP*** | GGACGGCTACAAGAACGACAC | TCATGCTGCATGACCGTGAT |
| (Smc00324) |  |  |
| *Polyribonucleotide nucleotidyl transferase* |  |  |
| ***nifD*** | TCCGACTTTCAGGAGAAGGA | CGATGTCGTCACCAATCAAG |
| *Nitrogenase molybdenum-iron protein alpha chain* |  |  |
| ***nifH*** | GTTATCACCTCGATCAACTTCCT | CGATGTAGATTTCCTGAGCCT |
| *Nitrogenase iron protein* |  |  |
| ***Thrxs1*** | TGTGTTGGTGGAATTCTTCG | AGCCACGAGTTTGTTTCGAC |
| *Thioredoxin s1* |  |  |
| ***Lb*** | GAGCGAAGAATTGAGCACTGCT | TGCCTTCTTAATTGCAGTTGCC |
| *Leghemoglobin* |  |  |
| ***NCR001*** | CACTTCTGACGCTAACTG | TGAACCTTCAATATACCACC |
| *Nodule-specific Cysteine-Rich peptide 001* |  |  |
| ***CP6*** | CCTGCTGCTACTATTGCTGGATATG | CACTCGCATCAATGGCTACGG |
| *Cysteine Protease 6* |  |  |
| ***VPE*** | CCAGGGGTTCTTGGTATGCCCG | ACTGCCAGATTCACATGCCTCCA |
| *Vascular Processing Enzyme* |  |  |
| ***PAP*** | TTTTGCACCGGATTCTTCAC | CCAACACCACTACCCAAGGA |
| *Purple Acid Phosphatase* |  |  |

**Table S2:** expression analysis of *pap* isoforms up regulated in nodules compared to roots. Pap isoforms were found in symbimics (https://iant.toulouse.inra.fr/symbimics/) website using “purple acid phosphatase” as a key word. Amongst the 52 isoforms, 5 were significantly more expressed in nodules than in roots. The pap Mt0031_00308 is significantly more expressed in zone III than in all the other zones. Numbers correspond to measures from nodule laser dissection (deseq-normalized-RNA-seq reads).

**Figure S1:** Growth of *Sinorhizobium meliloti* strains.

The growth of Rm2011, mutant strain SmgshB in Rm2011 background was monitored by measuring the OD_600_. Growth of SmgshB was complemented with GSH 2mM. At time zero, rhizobial cells were diluted with LB medium to an OD_600_ of 0.1. The data are the means from three experiments.

**
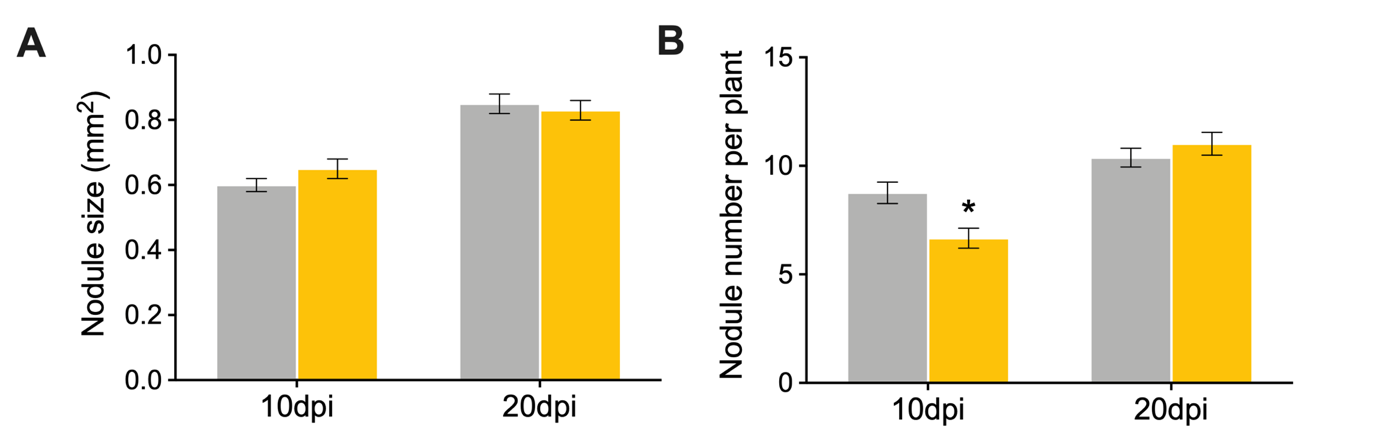
**

**Figure S2:** Nodule development at 10 and 20 dpi in WT (grey) and GshB mutant (yellow).

A. Nodule size (mm^2^). B. Nodule number per plant. Data (three biological samples) are reported as mean ± standard error. * indicates a statistically significant difference relative to the control (P <0,05; ARA, nodule number and Plant shoot weight were calculated with 15 plants and nodule surface was measured with 75 nodules).

**
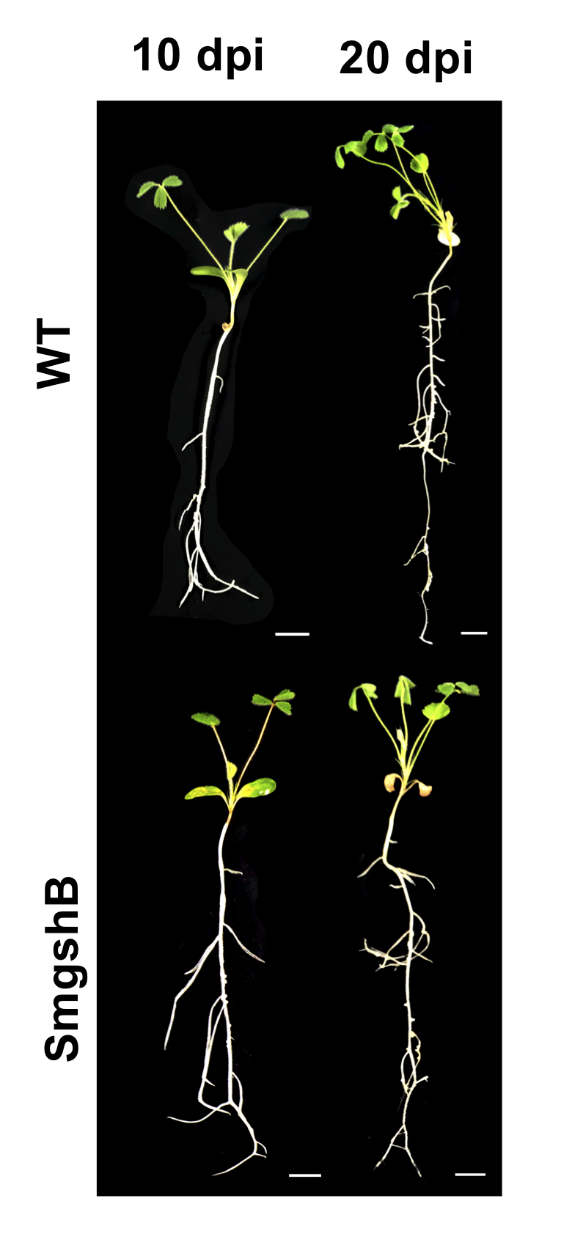
**

**Figure S3:** Plant development at 10 and 20 days post infection.

Plants were inoculated by *S. meliloti* 2011 (WT) and *S. meliloti* 2011 gshB mutant (SmgshB) strains. The image is representative of 15 plants. Scale bar = 1 cm
